# Material characterization of the Turkana Abarait

**DOI:** 10.1098/rsos.250883

**Published:** 2025-11-05

**Authors:** Kate Parkinson, Parvez Alam

**Affiliations:** ^1^ School of Engineering, The University of Edinburgh, Edinburgh, UK

**Keywords:** Turkana, Abarait, wrist blade, material characterization, African metallurgy, traditional African weapons

## Abstract

In this article, we research the material, mechanical, geometrical and chemical characteristics of the Turkana Abarait, a wrist blade used ubiquitously by Turkana people (both male and female) in north-western Kenya. To characterize the blades, we used a combination of three-dimensional scanning, scanning electron microscopy coupled with Image Analysis techniques, X-ray diffraction, X-ray fluorescence and Vickers hardness testing at HV30. We find that the blades are made from low-carbon bloomery iron, containing particulates of slag inclusions, or soot-based remnants, as well as trace elements of magnesium, sodium, aluminium, sulfur, phosphorus, chlorine, cobalt and potassium. While the soot particulates are likely derived from the incomplete refinement and non-uniform heating typical of pre-industrial forging methods, we deduce that the other trace elements originate from irons smelted from riverbed ore. We find that while blade edge angle does not differ between the blades (*p* > 0.05, 95% confidence), the blade edge widths are significantly different (*p* < 0.001, 99.9% confidence), indicating inconsistencies in the manufacturing processes. We find that there are mechanically significant differences in both inter- and intra-blade hardness values (*p* < 0.001, 99.9% confidence), adding to our proposition that Abarait blades are manufactured inconsistently. The blades are nevertheless fit for purpose, achieving a balance of hardness and ductility suited to their dual role as cutting tools and close-combat weapons.

## 1. Introduction

### 1.1. Cultural context

The Turkana region is in the north-west of Kenya, connecting East Africa with Sudan and Ethiopia. Turkana is bounded by the Rift Valley escarpment on the west and Lake Turkana on the east, Lake Turkana being the world’s largest permanent desert lake [1]. Spanning 68 233 km${,}^{2}$, Turkana is the second largest county in Kenya [2] and the Omo-Turkana region that extends into southern Ethiopia is well known as the ‘Cradle of Mankind’ due to the early human fossils found there [1]. The county’s landscape consists of low-lying open plains, scattered mountain ranges and seasonal rivers. Temperatures range from 20°C to 41°C, with a mean temperature of 30.5°C [3]. The central region is a desert environment with sparse vegetation, areas of shifting sand dunes and an average annual rainfall of approximately 200 mm.

Given the region’s harsh conditions, most of its terrain is unsuitable for growing crops, causing the majority of the population to rely on livestock. The Turkana people have traditionally been nomadic herders, moving with their cattle in response to rainfall and pasture availability. A 2018 Turkana County government report states that 45% of the working-age population still participates in this kind of small-scale agriculture or pastoralism [4]. Despite increasing urbanization and development initiatives, pastoralism remains central to Turkana’s economy and culture, providing food, income and social status, resulting in livestock holding deep cultural significance. Even their widely believed origin story traces the Turkana’s ancestral roots to following a lost bull: a journey that ultimately led them to settle in their present lands and form their community [5]. Beyond being a source of food, cattle play a vital role in social identity, status and personal expression among the Turkana and many other East African pastoralist societies [6]. Ownership of cattle is not only an economic asset but also a symbol of prestige and masculinity, with men using their herds to demonstrate wealth, generosity and influence within the community. A Turkana man can modify the appearance of a ‘favourite-ox’ through branding, horn manipulation or cuts to the ears and dewlap—practices that closely resemble those depicted in Neolithic rock art scenes. These modifications are not merely aesthetic but serve as markers of identity and lineage, reinforcing the deep historical continuity of cattle-related traditions in Turkana culture [6].

Among many pastoralist groups, the ears of cattle are used to display various types of ownership marks [7]. However, for decoration purposes, the Turkana implement a specific ear cut using a traditional wrist knife that most adult Turkana wear. The Abarait is a wrist blade used by the Turkana people as both a weapon and a utilitarian tool. Due to the high value the Turkana place on their livestock, they will often raid other communities to acquire more animals. Although this could be seen as theft from an external point of view, it is considered a perfectly acceptable traditional custom among Turkana [8] and an Abarait could be used for defence when raiding other pastoralist communities in northern Kenya. Structurally, an Abarait is a circular or elliptical blade made of metal, worn around the wrist, with a sharp outer edge and a central cutout lined with leather for comfortable wear.

Anthropologists have been studying the pastoral communities of Africa since the 1940s [9], and for many years, scientists have recognized north-west Kenya as a region of significant archaeological interest [10]. Despite this, little research has been conducted on the Abarait, a weapon and tool that remains in use today. Art dating back to 3000 BC [6] suggests a deep historical continuity in its function and use, but little is known about its manufacture or materials. Given its enduring cultural relevance, the Abarait should be examined not only as an artefact of the past but also as a functional tool that continues to shape Turkana traditions and practices in the present day. This study aims to provide insights into the cultural significance of the Abarait, enhancing understanding of its role in Turkana heritage.

### 1.2. Turkana Abarait

**Figure 1.**
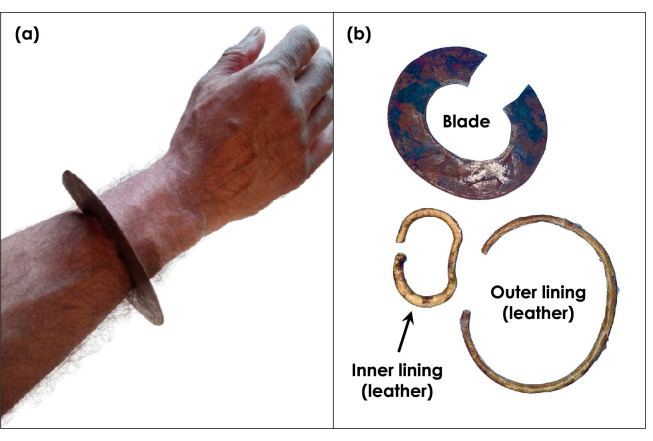
(a) Example of an Abarait worn on the wrist and (b) Abarait blade, the leather inner lining and the leather outer lining.

The Turkana Abarait holds both practical and symbolic significance within the Turkana community. The Turkana people express themselves through personal adornments, and their position in life is discernible by the types of ornaments or even the hairstyle they wear [11]. Unlike many African weapons that were used primarily for hunting or warfare, the Abarait serves multiple functions, including utility tasks, self-defence and status representation. The Abarait’s distinctive curved blade, worn on the wrist, allows the wielder to maintain full hand mobility, making it a versatile tool. Figure 1 shows (a) an example of an Abarait as worn on the wrist and (b) the different parts of the Abarait, including the blade, the inner leather lining and the outer leather lining. While the outer leather lining is mounted for safety when the blade is not in use, the inner lining can be used to enhance comfort.

The Abarait is commonly used to butcher livestock, slice meat and process hides, making it an essential tool for pastoralists. The curved design and keen edge are an effective means of making gashes that are long and deep [12] with minimal effort, which is particularly useful in the skinning of animals and the preparation of food. Additionally, the Abarait has historically served as a concealed self-defence weapon, offering protection in conflicts, cattle raids and personal disputes. Wood [13] recounts how the Abarait, then known as a ‘fighting bracelet’, was used in combat: ‘*Whenever the warrior comes to close quarters, he strips off the sheath, and, rushing in upon his adversary, strikes at the face with the sharp edge, or, flinging the left arm round him, cuts his naked body almost into pieces with rapid strokes of this terrible weapon*’ [12]. The compact, wrist-mounted design allows for quick deployment, making it ideal for close-quarters combat situations.

The Abarait can be worn by both sexes, but among men, it is often associated with warrior status and masculinity, serving as an indicator of bravery and combat readiness. In some cases, the Abarait is included in rites of passage, marking a young man’s transition to adulthood, similar to how spears and shields symbolize maturity in other East African societies [14]. Owning and wearing an Abarait may also be linked to a man’s wealth and social standing, as more elaborately crafted knives are often reserved for elders or respected warriors.

### 1.3. Traditional African metallurgy and blacksmithing

The craft of blade smithing holds a deeply respected place in African culture. The blacksmith’s workshop is often regarded as a sacred space, a place of magic, that holds ancient knowledge. Blacksmiths are seen as magicians who draw metal from raw stone and shape it with fire [15]. However, blacksmithing across many regions has experienced a marked decline, particularly in areas where formal education and the influence of Christianity have led younger generations away from traditional crafts. As early as 1980, it was observed that most blacksmiths were already middle-aged or elderly, with few young apprentices willing to continue the trade, largely due to diminishing belief in these ancestral obligations and ritual consequences [16].

Studies on African smelting furnaces have radiocarbon dated them to over 2500 years [17], with evidence of iron smelting found in regions such as Niger, Sudan and Kenya. The development of iron smelting and blacksmithing played a crucial role in shaping societies, influencing warfare, agriculture and trade. Archaeological evidence suggests there was no Bronze Age in sub-Saharan Africa [18], and apart from limited copper smelting in West Africa, the earliest evidence of metal working is in iron.

Unlike European blast furnaces, African smelters utilized bloomery furnaces, which produced wrought iron through the direct reduction of iron ore. The process involved charcoal-fuelled furnaces, often constructed from clay, where iron oxides were reduced to metal using a forced air system, typically powered by bellows or natural draught. These types of furnaces produce a spongy mass of iron, known as a bloom, which required extensive hammering to consolidate the metal [19].

Previous research suggests that iron ore was typically sourced locally, with many African communities having extensive knowledge of mineral deposits. The Turkana region, known for its arid landscapes and riverbeds rich in iron deposits [20], likely provided the raw materials needed for weapon production. The extent to which the Abarait was forged from locally sourced iron or from imported steel remains an open question that can be addressed using energy-dispersive X-ray spectroscopy (EDS) or X-ray diffraction (XRD) techniques [17].

Documentation shows the fabrication of weapons in African metallurgy often involved a combination of wrought iron and steel [21]. Forge-welding techniques were common, where high-carbon steel edges were welded onto softer iron cores, allowing for a balance of sharpness and durability. Differential hardening, where cutting edges were selectively hardened through quenching in water or oil, was also practised in various African societies [21]. This process ensured blades maintained a sharp cutting edge while retaining flexibility, preventing fractures upon impact. These methods align with findings from other archaeo-metallurgical studies, such as the analysis of medieval knives from Turkey, which demonstrated the widespread use of forge-welding, selective hardening and microstructural control in blade-making [22].

While historical African metallurgy has often been overshadowed in academic discourse, recent material analyses and experimental archaeology have highlighted its sophistication and innovation. Studies have revealed that African blacksmiths employed advanced techniques demonstrating a deep understanding of material properties long before the industrial age. Understanding these techniques provides essential context for analysing the Turkana Abarait, as its composition and forging methods may reflect regional metallurgical traditions, trade interactions or historic access to imported materials.

### 1.4. Artefact characterization methodologies

There is a lack of research on African tribal weaponry, particularly in comparison to the extensive metallurgical analyses conducted on Asian and European blades. Much of the existing research on African metalworking has focused on anthropological perspectives, leaving a gap in quantitative studies that examine material properties, forging techniques and structural performance using modern engineering tools. However, studies on other historical weapons and artefacts can provide insight on how to approach characterizing the Turkana Abarait. The characterization of other historical artefacts through metallurgical analysis and mechanical testing has provided valuable insights into traditional forging techniques and material properties. Okayasu *et al*. [23] examined samurai swords manufactured using the tatara steel making method [23], revealing that these weapons, despite numerous inclusions, exhibit high hardness and residual compressive stress due to fine-grained microstructures. The study highlights how variation in carbon content affects Vickers hardness and tensile strength. A similar relationship might be found within the Turkana Abarait.

Similarly, the archaeo-metallurgical study by Güder and Redford [22] analysed medieval knives from Kinet Höyük, Turkey, employing optical microscopy (OM), scanning electron microscopy with energy dispersive spectroscopy (SEM-EDS) and micro-hardness tests to investigate their composition and forging techniques [22]. Their findings indicate a diverse range of material compositions, from entirely steel blades to composite structures featuring forge-welded wrought iron and steel layers. These studies demonstrate how metallurgical techniques, including microstructural analysis and mechanical testing, can be applied to historical weaponry to reconstruct manufacturing practices, assess material performance and understand technological advancements of past societies. Using similar methodologies, the material composition and mechanical properties of the Turkana Abarait can be characterized.

XRD is a technique used in materials characterization to determine crystal structure, phase composition and residual stresses in metals and alloys [24]. This method is particularly useful in analysing historical weapons, where different heat treatments and forging techniques influence the formation of different phases. Studies including Yaso *et al*. [25] have used XRD to investigate residual stress profiles in samurai swords, revealing compressive stresses that enhance durability and toughness [25]. Similarly, applying XRD to the Turkana Abarait could provide insight into phase distribution, identifying whether differential hardening techniques were employed to enhance cutting efficiency and strength.

Energy dispersive X-ray spectroscopy (EDS), coupled with XRF analysis, allows for the detection of elemental composition in metallic artefacts [24]. This technique is particularly useful in identifying trace elements such as phosphorus, manganese or sulfur, which can influence the mechanical behaviour of a weapon. Studies on Viking swords and medieval knives [22] have shown that EDS can reveal variations in steel purity and slag inclusions, providing insights into the ore sources and smelting techniques used. Applying EDS to the Turkana Abarait would help distinguish whether it consists of high-purity iron or contains residual impurities from traditional smelting methods.

Reverse engineering techniques have proven valuable in archaeological and engineering research for analysing the structural and mechanical properties of artefacts. Moitinho de Almeida and Barceló [26] demonstrated how three-dimensional scanning can accurately capture the geometry of archaeological artefacts [26], enabling the development of precise CAD models that preserve intricate design details. These digital models facilitate quantitative analysis, overcoming limitations associated with traditional two-dimensional sketches or subjective visual assessments. Similarly, Neamțu and Comes [27] proposed a systematic methodology for digitizing archaeological artefacts, emphasizing not only the accuracy of three-dimensional models but also their portability [27].

### 1.5. Blade sharpness characterization

Blade sharpness is a concept that, despite its central importance to the function of edged tools, lacks a single, universally accepted definition. Although sharpness is commonly understood in practical terms as the ability of a blade to initiate a cut with minimal force or to produce a deeper incision at a given force, this does not easily translate into a measurable parameter. McCarthy [28] and Schuldt [29] both highlight that sharpness must consider not only the cutting force but also material deformation, tip geometry and wear resistance over time.

Wear resistance can be described as edge retention, the blade’s ability to maintain its edge shape. This ability preserves performance over time. Harder steels generally retain sharpness better but are also more brittle. Ductile steels allow for finer, more durable edges due to their resistance to micro-fracture during grinding [30].

The physical geometry of a blade is one of the most significant determinants of sharpness. A key factor is the edge radius (r), the radius of curvature at the very tip of the blade, which directly correlates with the force required to initiate a cut. Both theoretical and experimental studies confirm a proportional relationship between edge radius and cutting force: as r increases, the required force increases linearly [31].

The edge angle (ε) also influences blade performance. Marsot [30] demonstrated that the cutting force (*F*) increases with the tangent of half the edge angle, as described by:


(1.1) $$F\propto \tan\left(\frac{\varepsilon }{2}\right).$$

In practice, however, this relationship may be masked by other factors such as blade finish, cutting motion and frictional effects. The angle of a blade is still closely linked to its intended function, with sharper, narrower angles favouring precision and larger angles offering greater durability. For example, woodcarving knives are typically ground to a 20°−25° angle to maximize sharpness and control, which is essential for delicate, detailed work. In contrast, slicing, paring and filleting knives require a slightly broader 25°−30° angle to provide a tougher edge that withstands contact with bone or dense muscle tissue. The choice of blade angle reflects a compromise between sharpness and strength, tailored to the specific mechanical demands placed on the edge.

As studies of African tribal blades such as the Turkana Abarait remain rare, most existing work focuses on anthropological descriptions, but given its continued use as both a tool and a weapon, and its unique wrist-mounted geometry, the Abarait presents an important case for applying modern sharpness characterization techniques.

### 1.6. Justification of this study

Despite the rich history of African metallurgy, there remains a significant gap in engineering-based characterization of African tribal weaponry. As previously stated, while extensive research has been conducted on Asian and European weapons, relatively few studies have applied modern metallurgical and mechanical analysis to African weapons, particularly those still in use within indigenous communities like the Turkana Abarait. Understanding its material composition, forging techniques and mechanical properties is important for the preservation of indigenous knowledge, contextualizing African ironworking traditions and comparing them with global metallurgical practices. Observations on the decline of smelting and blacksmithing were made over four decades ago [16], it is reasonable to infer that traditional blacksmithing and smelting practices have diminished even further, placing many regional metallurgical traditions at risk of being lost entirely. Government restrictions on carrying traditional weapons have also contributed to the Abarait’s reduced visibility in public spaces, and Turkana blacksmiths say that the introduction of modern knives means the Abarait is no longer as desired [32]. These factors, paired with a shortage of materials, could lead to the Abarait disappearing from modern-day use before it has been properly studied. By integrating three-dimensional scanning, optical analysis and mechanical testing, this study will provide a quantitative and scientific approach to analysing the Abarait.

## 2. Methods

To address the lack of existing technical data on the Turkana Abarait, a comprehensive experimental methodology was developed to characterize the blade’s material composition, structure and sharpness using a combination of microscopy, spectroscopy and mechanical testing. Where possible, international standards and conventions were followed when designing these methodologies. Abaraits were procured from a Turkana blacksmith who made them by forging them into a circular shape through heating and hammering, using generationally bequeathed traditional blacksmith skills. The Turkana blacksmith was, at the time, based in Kanamkemer village, Lodwar Town, Turkana County, North Western Town, Kenya (3.1011° N, 35.6013° E).

### 2.1. Dimensional analysis

**Figure 2.**
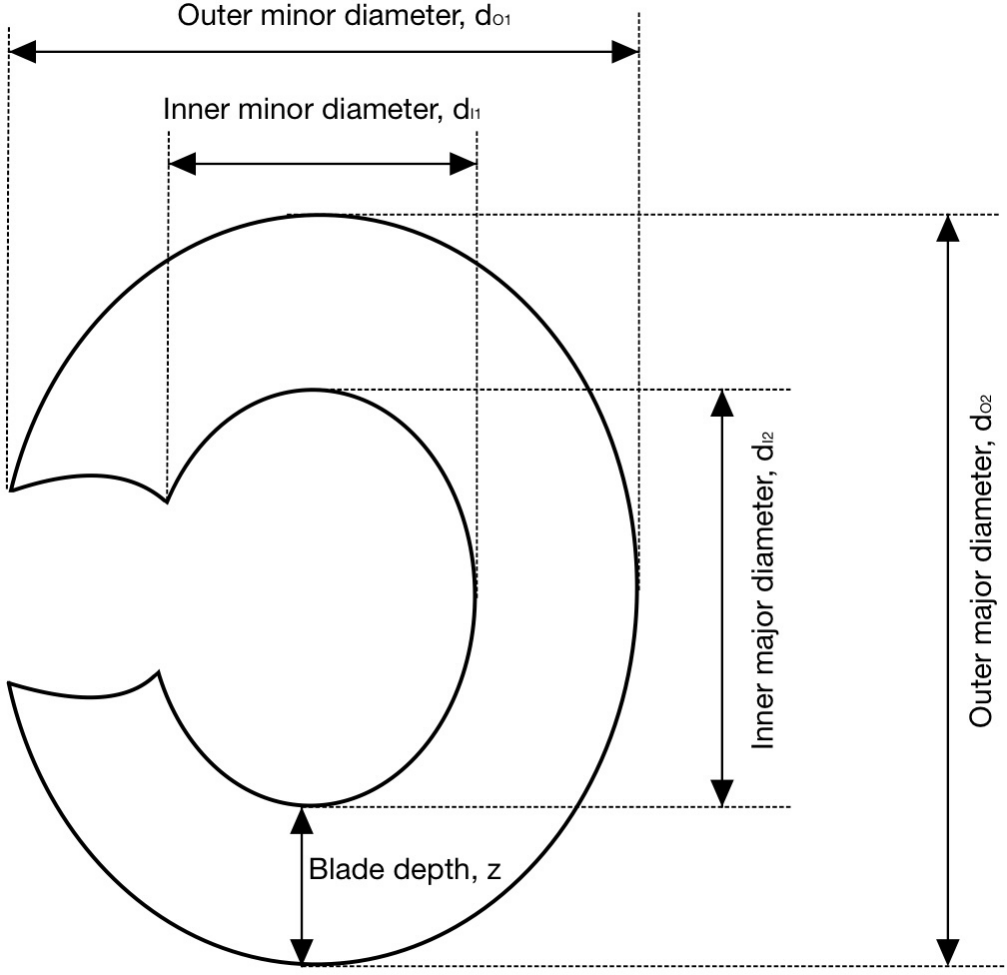
Diagram of Abarait blade showing the key dimensions measured. Parameters include the outer diameters ($d_{O1}$, $d_{O2}$) and inner wrist gap diameters ($d_{I1}$, $d_{I2}$), as well as the blade depth (z).

Initial measurements of each Abarait were taken to establish the main dimensions and geometries prior to any digital modelling or destructive testing. Digital callipers were used to measure the average blade depth, average thickness, the major and minor diameters and the dimensions of the central wrist gap (figure 2). Both blade depth (z) and thickness (t) were measured at five regularly spaced intervals to calculate mean values.

Each Abarait was then scanned using an EinScan HX, a three-dimensional scanner from Shining3D, allowing for the development of CAD models for future analysis, as well as the creation of permanent digital archives of the blades in their original, unaltered state. This archival step was critical for preserving the form and features of each artefact prior to any destructive sample preparation or testing.

### 2.2. Sample preparation

**Figure 3.**
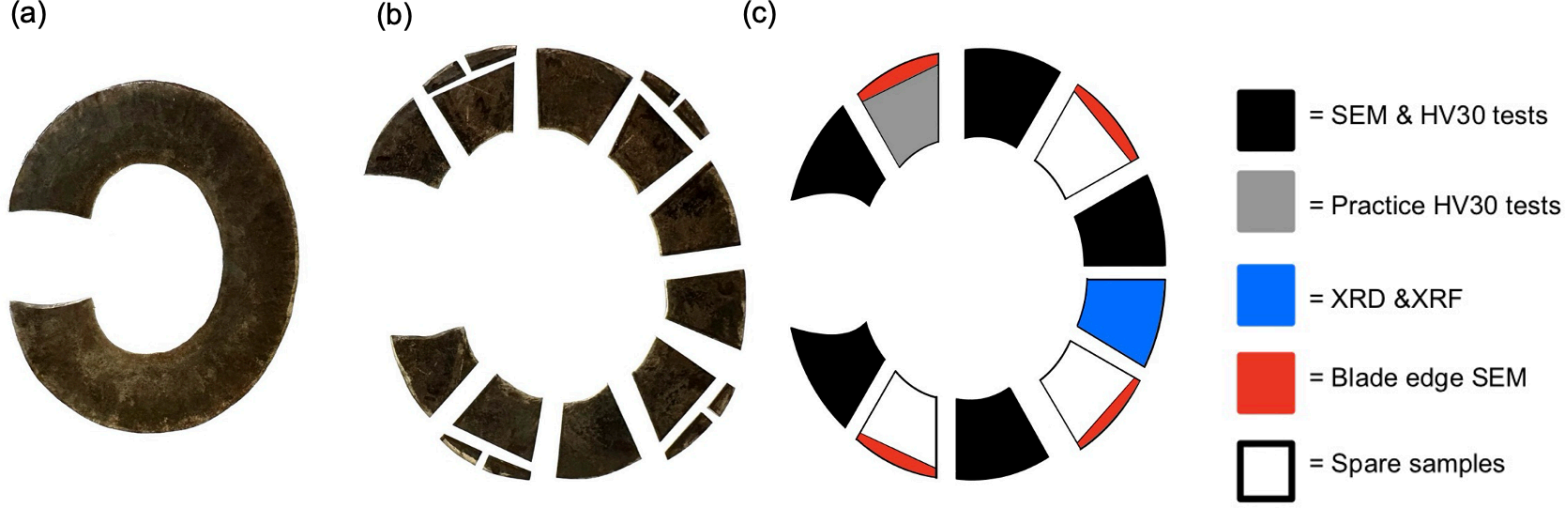
Progressive stages in the preparation of an Abarait blade. (a) A full intact Abarait blade. (b) A blade segmented into samples with some blade edges removed. (c) Schematic showing the use of each sample. Samples were assigned for various analyses: Vickers hardness testing (HV30), SEM, XRD and X-ray fluorescence (XRF). Spare samples were retained for potential future analysis.

Each Abarait blade was divided into 10 segments for further imaging and material characterization. The blades were cut using a sheet metal guillotine, which provided clean, consistent sections while minimizing thermal or mechanical deformation and ensuring representative sampling along the blade profile. As seen in figure 3, four segments from each blade had the edges cut off to fit in the SEM for edge geometry analysis.

The remaining segments were individually embedded in 40 mm diameter moulds using a two-part epoxy resin and left to cure for 24 to 48 h. Once cured, the resin-mounted samples were progressively polished following a modified ASTM E3-11 method that involved a series of silicon carbide papers (from P180 to P2500 grit) followed by polycrystalline diamond suspensions (3 µm and 1 µm) on polishing cloths. This process produced a smooth, reflective surface suitable for SEM and Vickers hardness testing. Destructive testing permanently alters and destroys the blades, and while this may in some cases be symbolically disruptive, this is not the case with the Abaraits. The production of empirical data and technical information on the blades is of importance as it helps to preserve their legacy through academic communication. Recognizing this importance, we sought and received permission to test the blades from the Turkana people, as is highlighted in the acknowledgements section.

### 2.3. Material characterization

Material characterization was carried out to investigate the composition and microstructural features of the Abarait blades. A Hitachi TM4000 Tabletop Scanning Electron Microscope (SEM) was used to capture high-resolution images of the polished surfaces at various magnifications. The microscope was operated at 15 kV using the standard Backscattered Electron (BSE) analysis mode to allow for microstructure and particle analysis.

The SEM images were further analysed using ImageJ FIJI software [33], which was designed to handle biological images but is well-suited for analysing SEM images of microstructures in materials science contexts, too. Each image was calibrated by setting a known scale using the SEM’s scale bar, allowing for accurate analysis. The images of the blade surface were then converted to binary (black and white) using a combination of automatic and manual thresholding techniques to clearly distinguish particles from the background. For the calculation of particle size and particle density, images taken at 2000× magnification were each split into six slices and values were found using automated measurement tools within FIJI, providing quantitative data on the microstructural characteristics of the blade.

In addition to SEM imaging, XRD and XRF analyses were performed on a different unpolished sample of the blade. XRD was employed to determine which phases were present, while XRF provided elemental composition data, allowing for an assessment of alloy content and potential trace elements.

### 2.4. Blade edge characterization

The blade edge of each blade was characterized to assess sharpness, geometry and potential functional intent. Key geometric parameters, edge angle (ε) and tip width (w), were measured for comparison with known standards in modern and traditional blades. To capture images of blade edges, the tabletop SEM was also operated at 15 kV but in standard secondary electron (SE) mode.

**Figure 4.**
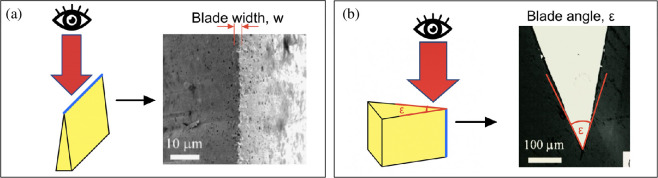
Illustration of sample orientation and image output for SEM of blade edge. Blue lines are the blade edge, red arrows indicate the direction of electron beam incidence and the eye shows the viewing direction. (a) Measurement of edge width (w) with sample positioned such that tip points upward. (b) Measurement of blade angle (ε), with sample oriented to expose cross-section geometry. Images originally from [34].

Images of each blade edge were imported into FIJI [33] and calibrated using the embedded scale bar. For each sample, blade width was measured at 10 evenly spaced intervals along the edge of the SEM image, to see any variation along the blade and calculate an average value. Figure 4a shows an example of a plan-view SEM image of a blade edge. Blade angle (ε) was calculated from the cross-section images (either 100× or 200× depending on clarity) by drawing tangents to the bevel faces and measuring the included angle at the tip apex (figure 4b).

### 2.5. Mechanical testing

**Figure 5.**
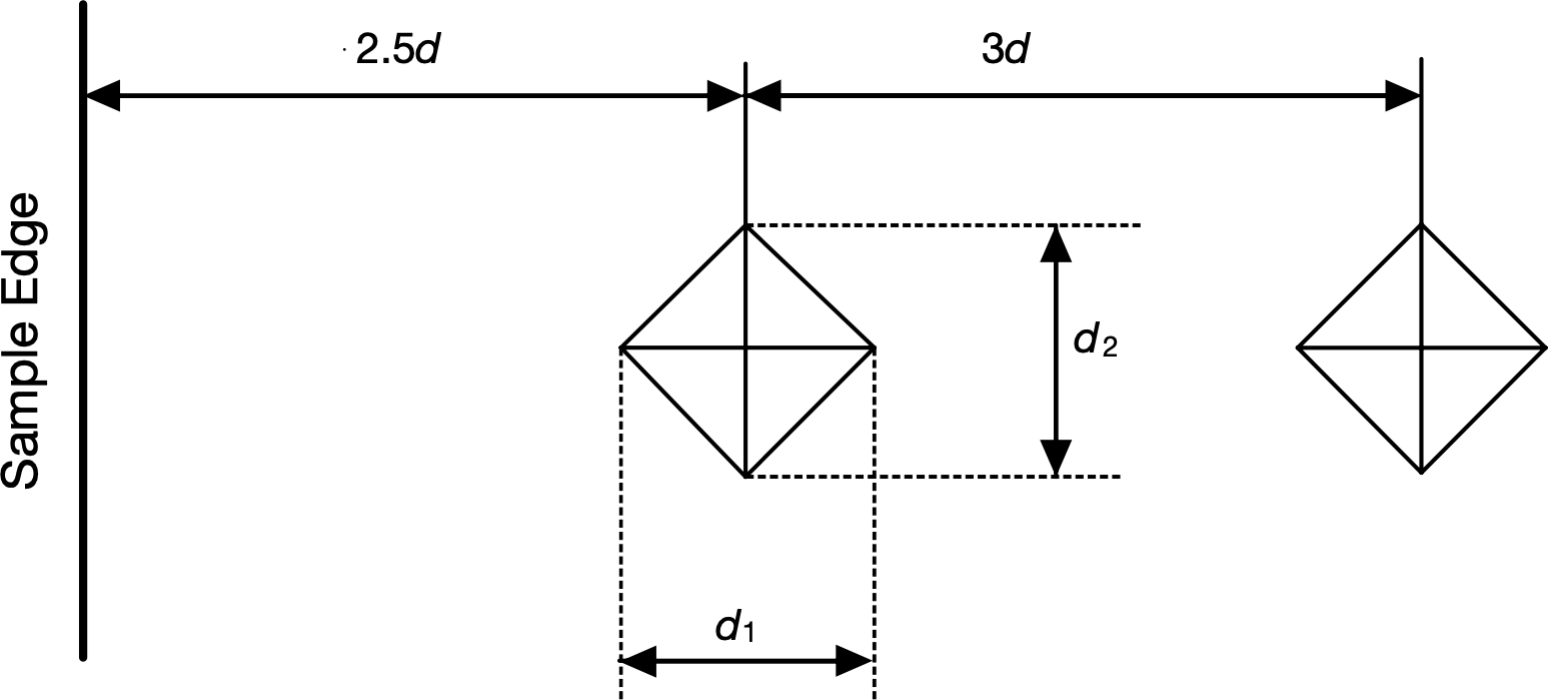
Minimum distance for Vickers indentations.

Vickers hardness testing (HV30) was conducted on polished samples using a microhardness tester in accordance with ISO international standards, to ensure consistency and reliability of results. HV30 indicates the Vickers hardness of the samples was measured using a 30 kilogram-force (*F* = 294.2 N) load, providing a standardized assessment of surface resistance to indentation. A practice indent was first made to determine the appropriate spacing between indents and minimum distance from sample edges required to avoid interaction effects. The ISO standard 6507-1 [35] states that there must be 2.5× the mean diagonal length (d) of an indent from the sample edge and 3×d between indents (figure 5).

**Figure 6.**
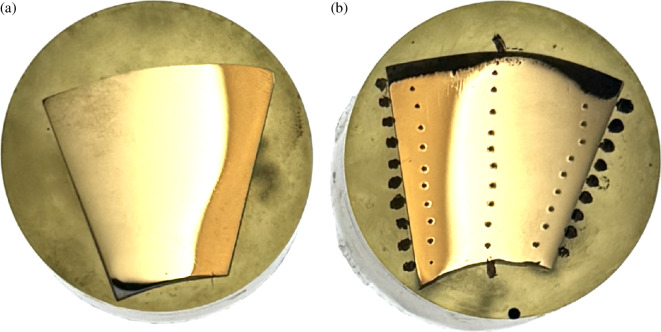
(a) Untested sample polished according to modified ASTM E3−11 method. (b) A Vickers hardness tested sample which shows indent pattern.

Each sample was tested with a series of 10 indents, spaced evenly from the inner to the outer edge of the blade. This sequence was repeated at three positions; the left edge, centre and right edge, providing approximately 30 HV values per sample (figure 6b). However, due to the curvature of some samples, it was not always possible to accommodate 10 indents without violating spacing constraints; in such cases, the maximum feasible number of indents was used. The samples were held with a vice during testing to ensure they did not move and that force was applied perpendicular to the flat surface.

After testing, a Dino-Lite digital AM4115FUT microscope, which emits light that excites at 375 nm and emits at 410 nm, was used to capture high-resolution images of each indent; these images were then analysed using ImageJ FIJI software. Each image was calibrated using a microscope scale slide, and the diagonal lengths of the indents ($d_{1}$ and $d_{2}$) were measured digitally to calculate Vickers hardness and tensile strength ($\sigma_{t}$) values.

Equation for Vickers hardness:


(2.1) $$HV=0.1891\cdot \frac{F}{d^{2}},$$

here


(2.2) $$d=\frac{d_{1}+d_{2}}{2}.$$

Relationship between Vickers hardness and tensile strength:


(2.3) $$HV=C\cdot \sigma_{t}.$$

In this study, C≈3 was used as it is an empirical relationship widely used in literature [36].

## 3. Results and analyses

### 3.1. Dimensional analysis

Dimensional measurements were conducted to assess the size and overall geometry of each Abarait blade prior to destructive testing or digital analysis. The results, summarized in table 1, show measurable variation between blade 1 (B1) and blade 2 (B2) across all recorded parameters.

**Table 1.** Summary of dimensions for blade 1 (B1) and blade 2 (B2), including outer diameters ($d_{O1},d_{O2}$), inner diameters ($d_{I1},d_{I2}$), mean blade depth (z) and mean blade thickness (t). Standard deviation (s.d.) values are provided for z and t based on n=5 measurements taken around the blade circumference.

	*d* ${,}_{O1}$	*d* ${,}_{O2}$	*d* ${,}_{I1}$	*d* ${,}_{I2}$	*n*	Mean *z*	s.d.	Mean *t*	s.d.
	(mm)	(mm)	(mm)	(mm)	—	(mm)	—	(mm)	—
B1	123.0	106.0	61.0	42.5	5	31.2	0.837	0.206	0.009
B2	118.5	99.0	63.5	44.5	5	27.7	0.837	0.182	0.022

Blade 1 was slightly larger, with an outer minor diameter ($d_{O1}$) of 123.0 mm and an outer major diameter ($d_{O2}$) of 106.0 mm, compared to 118.5 mm and 99.0 mm for blade 2, respectively. Similarly, the inner diameters ($d_{I1}$, $d_{I2}$) which correspond to the central wrist gap were marginally larger in B2, indicating subtle variation in user wrist size or construction. Measurements of blade depth (z) and thickness (t) were taken at five (n = 5) evenly spaced locations along the blade edge and averaged. Blade 1 was marginally deeper and thicker on average (table 1). Both blades exhibited identical standard deviations about the arithmetic means for blade depth, suggesting similar variation across measurement points. However, blade 2 showed a higher standard deviation in blade thickness, indicating a slightly greater inconsistency in thickness across its profile.

The dimensional measurements reveal subtle but consistent differences between the blades in both outer geometry and wrist gap dimensions. While these variations may initially appear to reflect random forging variability, they are more plausibly explained by the individualized and culturally informed production practices observed among Turkana blacksmiths. Given that Abarait blades are worn and used by both men and women, young or old, it is possible that the blades in this study were crafted with different intended users in mind.

Ethnographic accounts indicate that some smiths estimate dimensions by experience, while others adopt a more personalized method, using fibre or twine to measure the customer’s wrist before transferring this length to the metal bar during shaping [16]. This approach would account for the observed differences in inner diameters ($d_{I1}$ and $d_{I2}$), which likely correspond to wrist size. The variation between the blades indicates that blades were not made to any sizing standard, applicable therefore to a broad range of wrist sizes. The larger external dimensions and greater thickness of blade 1 may reflect a blade designed for a user requiring increased durability or reach, while blade 2’s smaller and thinner form could indicate use by a smaller-bodied individual or someone prioritizing speed and mobility.

Overall, the dimensional variability observed supports the interpretation that Abarait blades were not standardized tools, but rather custom-forged artefacts reflecting a combination of functional, ergonomic and cultural considerations. These findings underline the importance of viewing the Abarait not only as a weapon or tool, but as a personalized object of material culture shaped by both individual identity and community craft practices.

### 3.2. Material characterization

#### 3.2.1. Optical analysis

**Figure 7.**
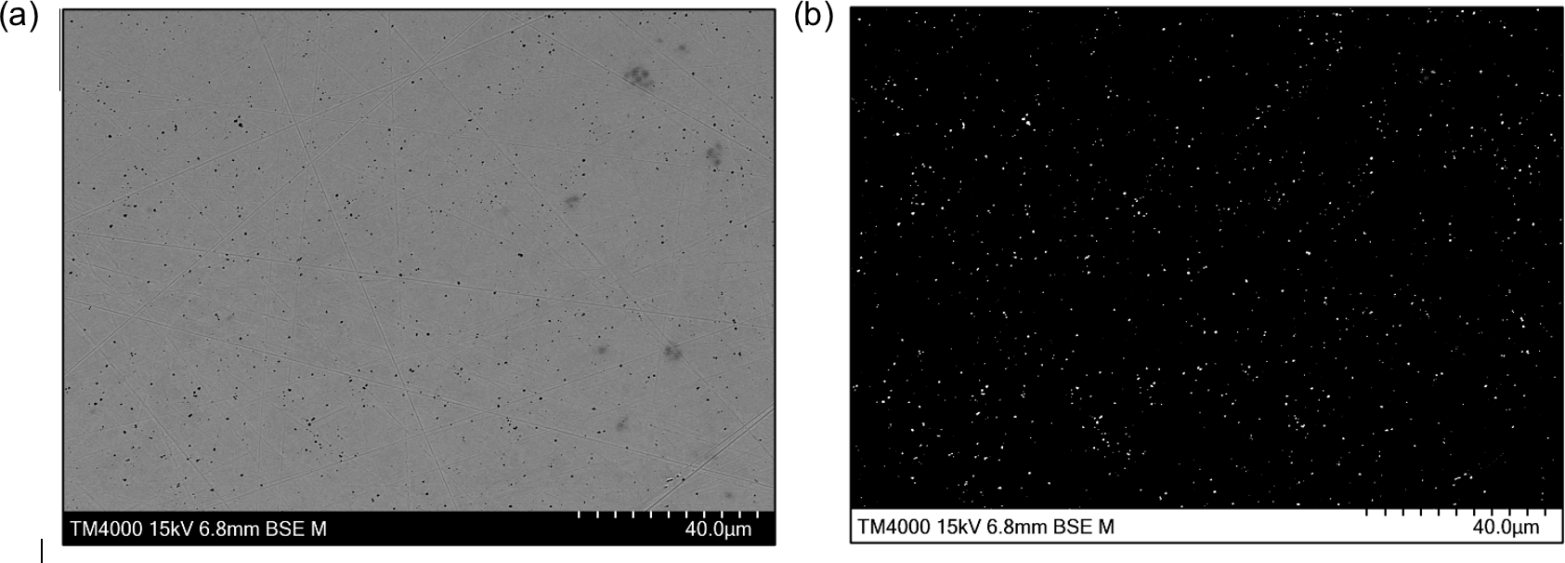
Example of a 2000× magnification SEM image (a) before and (b) after applying binary threshold in ImageJ FIJI to show particles.

Initial SEM images showed there were no visible grain boundaries. This is because the samples were polished, not etched. However, the presence of particulates in the metal was visible in the SEM images. Representative images were captured at 2000× from polished samples and processed using FIJI to apply a binary threshold for particle identification. Figure 7 shows an original SEM image and the corresponding binary version, clearly highlighting discrete surface features.

Particle size and particle density were calculated for five samples from blade 1 and five from blade 2, the descriptive statistics are summarized in table 2. In this context, particle density refers to the number of discrete particles identified within a given cross-sectional area of the blade surface, expressed as particles per square micrometre (particles µm^−2^). FIJI has an automated feature for particle size and quantity, but particle density was calculated using equation (3.1):


(3.1) $$\begin{array}{lcr} & \text{Particle Density}=\frac{\text{Number of Particles in Slice}}{\text{Area of Slice}} & \end{array}$$

**Table 2.** Statistics for particle size and particle density for blade 1 (B1) and blade 2 (B2). For particle size, n refers to the number of particles visible in binary SEM images, and for particle density, n is the number of slices analysed to calculate other values (6 slices per 6 images to a total n=36).

		*n*	mean	median	s.d.	min	max
particle size (µm${,}^{2}$)	B1	9858	0.0525	0.0190	0.085	9.41E−04	3.349
	B2	11 022	0.0576	0.0110	0.268	0.00200	12.30
particle density (particles µm^−^${,}^{2}$)	B1	36	0.149	0.122	0.093	0.0516	0.419
	B2	36	0.119	0.105	0.101	0.00273	0.449

**Figure 8.**
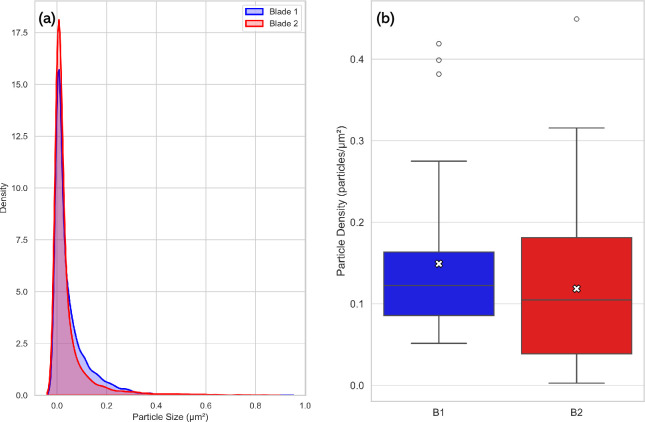
(a) Comparison of probability density functions showing distribution of particle sizes for blades 1 and 2. Outliers beyond ±3 s.d. were removed prior to plotting. (b) Comparison of box plots showing complete particle density distributions for B1 and B2.

To further explore differences between the blades, comparative plots were generated (figure 8), showing the distributions of particle sizes (figure 8a) and densities (figure 8b). While both blades exhibited overlapping ranges, blade 2 showed a slightly higher variability in particle size and demonstrated a greater particle density. The particle density box plots in figure 8b provide a clear visual comparison between the blades. It should be noted that there were many outliers in the particle size distributions, and figure 8a was constructed removing outliers, which were determined using a *z*-score analysis, $z=\frac{x-\mu }{SD}$, where x represents the observed data, μ represents the mean for the sample set and SD is the standard deviation of the sample set. Values where z≤−3 or z≥3 indicate sample set values ± 3s.d. from the arithmetic mean, and these were removed as statistical outliers.

Table 3 provides insight into the number and size range of particles observed in both blades. The probability density function in figure 8a shows the distributions for both blades are skewed towards smaller particles, showing that particles were mostly within a 0 to 1 micrometre range. Blade 1 and blade 2 had the same modal particle size (0.002 µm${,}^{2}$), indicating that the particle size that occurs the most frequently was identical between samples, despite potential differences in overall distribution or density.

**Table 3.** Summary of mean particle size, s.d., s.e., coefficient of variation (CoV) and significance tests for blade 1 and blade 2, where n refers to the number of particles visible in thresholded SEM image.

	mean	*n*	s.d.	s.e.	CoV	ANOVA *p*‐value	two-tailed *t*‐test *p*‐value
	(µm^2^)	—	—	—	—	—	—
B1	0.0525	9858	0.0845	0.00053	1.61%	$4.26\times 10^{-204}$	0.059
B2	0.0576	11 022	0.2685	0.00055	4.66%	$8.95\times 10^{-70}$	

The microstructural analysis of the Abarait blades offers insight into the metallurgical processes used in production. SEM imaging revealed the presence of dispersed particles within the metal matrix, interpreted as slag inclusions or soot-based remnants, features consistent with traditional bloomery ironworking. These inclusions point to incomplete refinement and non-uniform heating typical of pre-industrial forging methods. They were believed to be soot particles, which can range in size from 10 to 30 nanometres (nm), but some studies show a wider range of sizes from 0.001 µm (1 nm) to 2 µm as soot particles are formed during incomplete combustion and are often larger, agglomerated nanoparticles or smaller particulates of the components of soot [37].

While both blades showed similar mean particle sizes (table 3), blade 2 exhibited significantly greater variance and a higher coefficient of variation, indicating a less homogeneous structure. This suggests more variable thermal or mechanical treatment during its manufacture, perhaps due to inconsistent exposure to heat during forging or cooling. The fact that blade 1 and blade 2 share the same modal particle size suggests a similarity in the dominant particle type or formation process, and figure 8 shows the majority of particles are within the 0 to 1 µm range, which is consistent with the known dimensions of soot particles.

Similarly, although blade 1 showed slightly higher average particle density (table 4), the difference was not statistically significant (*t*‐test *p*‐value =0.186). However, the highly significant ANOVA results for both blades (p<0.05 corresponding to a confidence level of 95%) confirm considerable intra-blade variability, again reinforcing the interpretation of manual fabrication techniques with limited control over microstructure. The presence, size range and distribution of particles are consistent with exposure to combustion environments, potentially during fabrication, maintenance or ritualistic use.

**Table 4.** Summary of mean particle density, s.d., s.e., coefficient of variation (CoV) and significance tests for blade 1 and blade 2, where n refers to the number of slices analysed to calculate other values.

	mean	*n*	s.d.	s.e.	CoV	ANOVA *p*‐value	two-tailed *t*‐test *p*‐value
	(particles µm^−2^)		—	—	—	—	—
B1	0.149	36	0.093	0.025	62.34%	$1.23\times 10^{-5}$	0.186
B2	0.135	36	0.101	0.022	75.30%	$1.22\times 10^{-10}$	

#### 3.2.2. Spectroscopy

**Figure 9.**
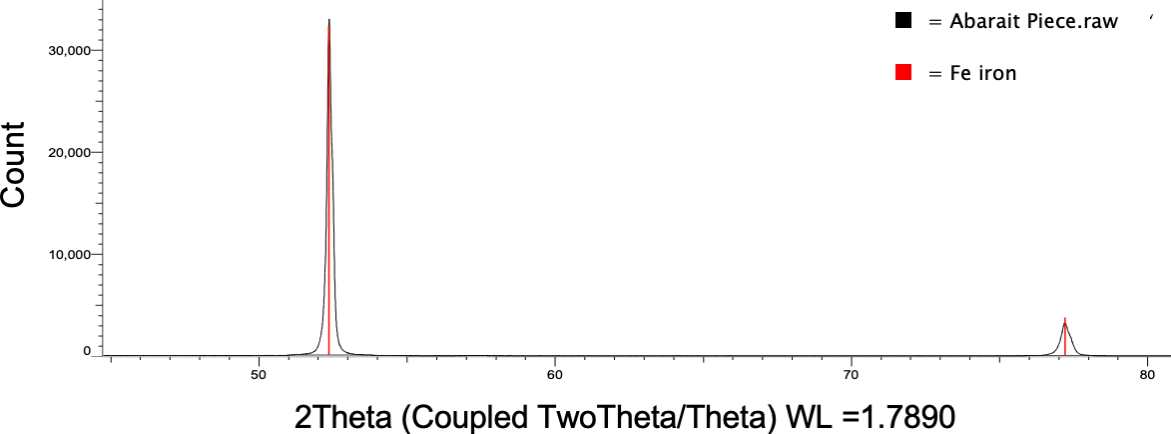
XRD spectrum of Abarait sample (black) showing a peak of 30 000+ counts at 52.5° 2θ and a peak of 4000+ counts at 77° 2θ. Peak positions correspond with overlaid reference pattern for iron (red).

To characterize both elemental composition and phase structure of the Abarait blades, XRD and XRF analyses were conducted. The XRD spectrum (figure 9) reveals sharp diffraction peaks that closely match the reference profile for pure iron (Fe), indicating the primary crystalline phase of the blade is metallic iron. The sharp, strong peak around 52.5° 2θ and the smaller one near 77° 2θ line up with known XRD reflections for α-Fe (body-centred cubic iron) [38]. No secondary phases were detected within the resolution of the scan, suggesting a relatively homogenous iron matrix consistent with traditional forging. There is no indication of significant cementite or pearlite content, suggesting that any heat treatment applied was insufficient to drive carbon diffusion to levels typical of steel or cast iron and further implies either minimal quenching or a lack of intentional carburization during manufacture. These results support the conclusion that the blades were made using bloomery-derived iron, with limited refinement and without the addition of modern alloying or hardening practices.

Elemental analysis using XRF identified the presence of iron (Fe) and several minor elements, including magnesium (Mg), sodium (Na), aluminium (Al), sulfur (S), phosphorus (P), chlorine (Cl), cobalt (Co) and potassium (K). The presence of these elements does not indicate intentional alloying and is instead interpreted as naturally occurring impurities introduced during the material’s extraction or processing. These impurities align with typical profiles of iron smelted from surface deposits or riverbed ore, further supported by the geological characteristics of the Turkana region. This geochemical profile furthermore aligns with iron that may have been sourced from sediment-rich riverbeds or collected and processed in brackish water environments. This is particularly relevant given that the Turkana people inhabit the region surrounding Lake Turkana, a brackish and alkaline lake in northwestern Kenya [39]. Traditional ironworking in parts of Kenya has historically relied on surface and riverbed deposits [16], where water chemistry and mineral content can introduce a range of trace elements into smelted iron. These findings suggest that the iron used in the manufacture of the Abarait may have originated from locally sourced materials that inherently reflect the mineralogy of the surrounding environment, offering insight into the regional sourcing and environmental conditions associated with the Abarait’s production.

Overall, our findings indicate that the Abarait blades were likely fabricated from low-carbon, ferritic iron produced using traditional bloomery smelting techniques. The presence of microstructural impurities, high variability in particle characteristics and the absence of alloying elements or phase transitions supports the view that the Turkana blacksmiths achieved functionally effective tools despite working within significant material and technological limitations.

### 3.3. Blade edge characterization

**Figure 10.**
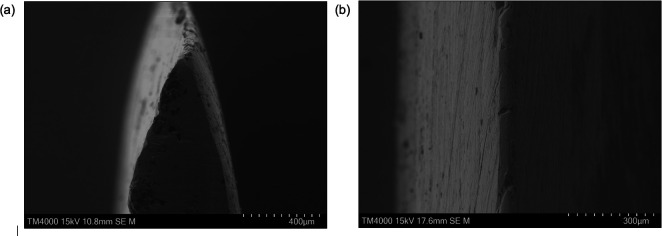
(a) Example of an SEM image used to calculate blade angle (ε). (b) Example of an SEM image used to calculate blade width (w).

To evaluate the sharpness of the Abaraits, the blade edge angle and width were measured from SEM images (cf. Figure 10 taken at 15 kV in SE mode). A summary of the results is provided in table 5.

**Table 5.** Summary of edge angle (ε) and edge width (w) for blade 1 (B1) and blade 2 (B2), where $n_{\varepsilon }$ is the number of angle measurements used to calculate mean edge angle and $n_{w}$ is the number of width measurements used to calculate mean blade width value.

	*n* ${,}_{\varepsilon }$	mean ε	ε range	s.d.	*n* ${,}_{w}$	mean width	width range	s.d.
	—	( ${,}^{\circ }$ )	( ${,}^{\circ }$ )	—	—	( μ m)	( μ m)	—
**B1**	4	44.0	30.6−49.7	9.03	40	32.4	13.9−72.1	12.0
**B2**	4	31.9	27.0−34.8	3.41	40	118.7	20.7−245.3	87.3

Blade 1 had a higher mean edge angle (44.0${,}^{\circ }$) than blade 2 (31.9${,}^{\circ }$), with a wider spread (SD = 9.03 versus 3.41), indicating that blade 2 had a sharper and less variable cutting edge. The wide blade angles, ranging from 30.6${,}^{\circ }$ to 49.7${,}^{\circ }$ for blade 1 and 27.0${,}^{\circ }$−34.8${,}^{\circ }$ for blade 2, suggest that these knives were not optimized for precision slicing but prioritized durability and impact resistance.

For each blade, width measurements were taken at 10 evenly spaced locations along the plan view image of the cutting edge to find an average width. In contrast to our findings on blade angles, blade 2 had a larger average edge width (118.7 µm) compared with blade 1 (32.4 µm), accompanied by greater variability (s.d. = 87.3 µm versus 12.0 µm). The large range of edge widths indicated a lot of variation in both edges, also visible in SEM images. This likely results from differing sharpening practices, material inconsistencies or use-related wear.

In addition to geometric measurements, qualitative features were also observed. SEM images revealed clear signs of surface wear, edge chipping and the presence of inclusions embedded near the cutting edge. These features suggest prolonged use or exposure to hard materials and are consistent with forging techniques that may not fully refine the microstructure. Furthermore, in several sections, the blade edge exhibited a slight curvature rather than forming a perfectly straight tip. This curvature may have resulted from hand-forging asymmetries or long-term deformation due to repeated use and sharpening.

Blade edge analysis provided a means of evaluating the functional performance of the Abarait as both a cutting tool and close-combat weapon. SEM imaging enabled quantitative assessment of the blade angle and edge width, which serve as primary indicators of sharpness and durability. Compared to other blade types, the Abarait edge geometry aligns more closely with utility or butchering tools than precision blades. For example, modern filleting or slicing knives often have edge angles between $20^{\circ }$ and $30^{\circ }$, while wood carving tools may fall below $20^{\circ }$ to allow for fine detail work. In contrast, the Abarait’s broader edge angles, particularly in blade 1 which averaged $44^{\circ }$, are more comparable to those of hunting knives or traditional butchering blades, where durability takes precedence over initial cutting force [28,40]. Additionally, the *t*‐test *p*-value of 0.066 (cf. table 6) indicates that the difference in angle between blades is not statistically significant, suggesting the design emphasis was on durability over precision cutting for both blades.

**Table 6.** Summary of mean, s.d., s.e., CoV and significance tests for blade edge characteristics.

		mean	*n*	s.d.	s.e.	CoV	two-tailed *t*‐test *p*‐value
edge angle	B1	44.03	4	9.03	22.02	20.5%	0.06602
( ε , ${,}^{\circ }$ )	B2	31.91	4	3.41	15.96	10.7%	
edge width	B1	32.43	40	12.03	5.13	42.6%	$2.536\times 10^{-7}$
( w , μm )	B2	118.67	40	87.26	18.76	73.5%	

This design choice is functionally consistent with the Abarait’s purpose. As a weapon, the blade required enough mass and edge strength to inflict injury without rapidly dulling or fracturing. As a utilitarian tool, particularly for animal skinning and meat preparation, edge retention and resistance to deformation were likely more valuable than scalpel-like sharpness. The rounded profiles and minor edge chipping observed under SEM further support the hypothesis of repeated field use, where edge degradation was expected and managed through routine re-sharpening.

The curvature and slight asymmetry of the edge profile also suggest hand-forging and manual grinding, lacking the controlled symmetry found in factory-produced blades. Variability in edge width, shown by high coefficient of variation values (42.6% and 73.5%), is presumably due to the handcrafted nature of production, where uniformity would have been limited by available tools and working conditions. The difference in edge width between blade 1 and blade 2 was found to be highly statistically significant with p<0.001 ($2.536\times 10^{-7}$) corresponding to a confidence level of 99.9%, table 6, confirming a clear distinction in sharpening or wear characteristics. These features are consistent with the findings of studies on traditional Kenyan blacksmithing methods, in which tools were often shaped using locally available stones as anvils and hammers, rather than purpose-made equipment. Chisels were also rarely used, unless repurposed from discarded objects such as axe heads [16]. This unrefined method of shaping metal naturally leads to minor inconsistencies in blade geometry, yet it also highlights the ingenuity of local craftsmen working with limited tools and materials.

Taken together, the edge characteristics of the Abarait reflect a pragmatic optimization of geometry and toughness, tailored to its cultural role and environmental context. Its geometry prioritizes resilience and functional longevity over cutting finesse. Which is an appropriate compromise for a blade intended to perform reliably across a wide range of pastoral tasks and combative scenarios.

### 3.4. Mechanical testing

**Figure 11.**
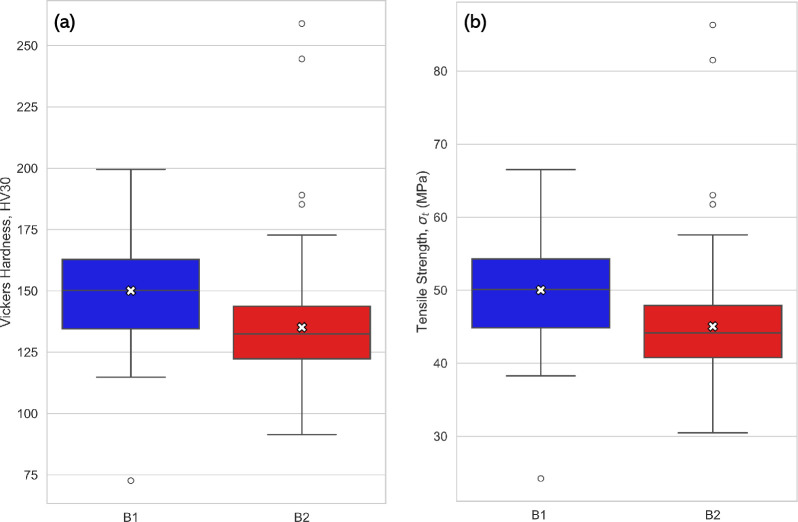
(a) Comparison of HV distributions for B1 and B2. (b) Comparison of tensile strength distributions for B1 and B2.

Mechanical tests were performed to evaluate the hardness and tensile strength of the two blades. These properties are critical indicators of edge retention, durability and overall mechanical performance of the Abarait blade. Table 7 summarizes the descriptive statistics, providing an overview of the central tendencies and variability observed across both samples, and figure 11 shows box plots of Vickers hardness and tensile strength for blades 1 and 2.

**Table 7.** Statistics for Vickers hardness and tensile strength for blade 1 (B1) and blade 2 (B2), where n is the number of HV test measurements used to calculate other statistics.

		*n*	mean	median	s.d.	min	max
Vickers hardness (HV30)	B1	145	150.1	150.3	20.2	72.7	199.6
	B2	150	135.1	132.5	22.4	91.44	258.96
tensile strength ( $\sigma_{t}$ , MPa)	B1	145	50.0	50.1	6.7	24.2	66.5
	B2	150	45.0	44.1	22.4	30.5	86.3

B1 exhibited higher hardness values, with a mean HV30 of 150.1 compared with 135.1 for B2. Tensile strength was calculated from Vickers hardness values (cf. Equation (2.3) where C=3), therefore, they follow a similar trend; B1 demonstrated a higher average ultimate tensile strength compared to B2. Alongside hardness data, B1’s higher tensile strength implies a greater load-bearing capacity, which is important for resisting fracture during impact, as implied by the general law $K_{IC}=\sigma_{t}\sqrt{\pi a}$, where $K_{IC}$ is the fracture toughness, $\sigma_{t}$ is the tensile strength and a is the crack length. Using the approximation $\sigma_{t}=\text{HV}/C$ with C=3, these values correspond to estimated tensile strengths of approximately 50 MPa for B1 and 45 MPa for B2. These figures align with expected mechanical behaviour of low-carbon, ductile wrought iron, which typically exhibits tensile strengths between 40 and 60 MPa and hardness values in the range of 100−160 HV30, depending on processing conditions, slag inclusions and carbon content.

**Figure 12.**
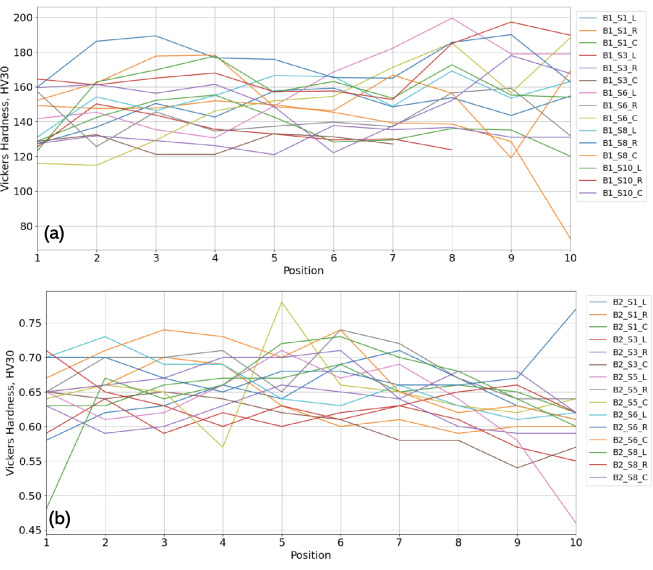
(a) Variation in HV30 from inner to outer edge for B1. (b) Variation in HV30 from inner to outer edge for B2. Where position 1 is the innermost HV test and position 10 is the outermost.

Several outliers were observed in both samples, particularly in B2, which had values exceeding 200 HV30 (figure 11). There was a higher standard deviation observed in B1’s hardness measurements than B2’s, suggesting a more pronounced variation across the blade. This may correlate with manual forging methods and thermal gradients. To investigate whether mechanical properties varied radially across the blade, line graphs were generated for each of the 15 datasets, showing the variation in Vickers hardness from the outer edge to the inner edge (figure 12). Although the visual analysis of these line plots revealed no consistent pattern in hardness distribution, further statistical analyses were performed to test for internal variation.

Significant variability was observed across and within samples (table 8). One-way analysis of variance (ANOVA) tests were applied to the data sets for each blade to assess the intra-blade hardness variation. The results indicated a statistically significant difference among the groups for blade 1, $p=8.77\times 10^{-12}$ (n=145) and for blade 2, $p=5.26\times 10^{-4}$ (n=150), confirming non-uniform hardness distributions as p<0.001 for both, corresponding to a confidence level of 99.9%. This is characteristic of blades forged using traditional methods, where localized cooling rates and manual hammering introduce thermal and structural heterogeneities. The smaller *p*-value for blade 1 suggests a greater degree of variation in hardness across its structure, possibly due to more variable thermal exposure during forging or differing cooling rates around the blade.

**Table 8.** Summary of HV, s.d., s.e., CoV and significance tests for blade 1 and blade 2, where n refers to the number of HV test measurements used to calculate other values.

	mean	*n*	s.d.	s.e.	CoV	ANOVA *p*‐value	two-tail *t*‐test *p*‐value
	(HV30)	—	—	—	—	—	—
B1	150	145	20.2	12.5	13.5%	$8.77\times 10^{-12}$	$5.24\times 10^{-9}$
B2	135	150	22.4	11.0	16.5%	$5.26\times 10^{-4}$	

Figure 12 reveals that no consistent radial trend in hardness was detected across either blade, suggesting that any differential treatment such as selective edge quenching was either minimal or not successfully retained in the material. This lack of directional patterning further supports the hypothesis of bloomery-based ironworking, where precise thermal control is difficult to achieve. Furthermore, a two-sample *t*‐test comparing the overall hardness between blade 1 and blade 2, table 8, revealed a statistically very significant difference between the two blades as p<0.001 ($5.24\times 10^{-9}$) corresponding to a confidence level of 99.9%.

The mechanical behaviour observed reflects the performance requirements of the Abarait: sufficiently hard to retain an edge, but ductile enough to absorb shock and resist fracture. The relatively low tensile strength compared to modern steels is compensated by the Abarait’s intended use context, where ease of production and resharpening were likely prioritized over edge retention or precision cutting. Overall, our results suggest that both blades were produced with practical performance in mind, achieving a balance of hardness and ductility suited to their dual role as cutting tools and close-combat weapons. The data reinforces the artisanal nature of Abarait production, in which functional adequacy was achieved despite the inherent variability of pre-industrial iron processing.

## 4. Discussion

### 4.1. Integration of results and context

The experimental characterization of the Abarait blades reveals a convergence of material ingenuity and cultural adaptation. When considering material composition, edge geometry and mechanical performance, the blades appear to be tailored to enable their dual use as weapons and utilitarian tools. Unlike modern industrial blades engineered for uniformity and specialized tasks, the Abarait embodies a broader functionality to meet the needs of pastoral life while retaining symbolic value. This convergence of cultural and engineering priorities is reflected in the consistent themes across all datasets: microstructural heterogeneity, moderate hardness, resilient geometry and visible signs of manual production. These features point not to technological inadequacy, but to an adaptive manufacturing strategy grounded in available materials, traditional knowledge and multifunctional demands.

### 4.2. Manufacturing insights and regional practices

The presence of α-Fe (ferrite) as the sole detectable phase in XRD analysis and the absence of cementite or pearlite confirms the use of low-carbon bloomery iron. This aligns with regional metallurgical practices across sub-Saharan Africa, where direct-reduction furnaces produced malleable iron blooms that were worked mechanically into blades and tools [16,17]. The lack of microstructural refinement beyond the ferritic phase suggests minimal or no quenching or carburization, which would have otherwise introduced harder microstructures typical of steel.

Trace elements such as sodium, chlorine and magnesium detected in XRF further support the use of local ore sources likely from brackish riverbeds or sediment-rich deposits surrounding Lake Turkana [20]. These impurities are consistent with the chemical signatures of iron extracted and smelted under open-air or clay furnace conditions with limited purification.

The observed microstructural variability, evident in particle size and density differences across blades, indicates a lack of thermal uniformity during forging. Blade 2, for instance, exhibited a higher coefficient of variation in particle size, likely due to less consistent forging temperatures or uneven working. These results support ethnographic accounts that traditional Turkana blacksmiths used basic hammering techniques, with anvils and hammers often fashioned from local stones and tools scavenged from prior materials such as discarded axe heads [16].

The asymmetry and slight curvature observed along the blade edges reinforce this interpretation, reflecting a hand-crafted process with limited geometric control. However, these inconsistencies do not imply poor workmanship, instead highlighting a method of production adapted to resource limitations and prioritized for function, not precision. Ethnographic accounts describe how blacksmiths sometimes use a piece of fibre or twine to measure a customer’s wrist before forging the blade [16], which may be an explanation for variations in inner diameter measurements. Given that Abaraits are used by a broad demographic, both young and old, male and female, this dimensional flexibility may reflect ergonomic adaptation as well as the decentralised, small-scale production by the Turkana smithie in Kanamkemer village from whom the blades were sourced.

### 4.3. Functional performance and use patterns

Blade 2’s narrower edge angle and sharper tip geometry suggest that it may have been kept for finer tasks, was either more recently sharpened, or just experienced less wear. Blade 1, by contrast, displayed a broader cutting edge and greater mechanical variability. Despite this, both blades fall well within the angular and edge-width range of robust utility knives rather than precision instruments [40]. This reinforces their role as practical tools rather than solely traditional or aesthetic artefacts and suggests a design prioritization of edge durability over acute sharpness.

Mechanical testing supports this interpretation. Both blades demonstrated hardness and tensile strength values consistent with ductile, low-carbon iron; sufficient for resisting plastic deformation under typical use, but soft enough to allow for easy re-sharpening. The values measured (mean HV30 of 150.1 for B1 and 135.1 for B2) are in line with historical iron tools forged without advanced alloying or heat treatment techniques.

The lack of a consistent radial trend in hardness and the significant intra-blade variability (as confirmed by ANOVA tests) further support the hypothesis of bloomery forging without differential treatment. In traditional Japanese katanas or medieval European blades, mechanical properties often vary deliberately across the blade, with hardened edges and softer cores introduced through quenching or layering [23]. No such pattern emerged in the Abarait, underscoring its production by more straightforward, less thermally differentiated methods.

Despite these limitations, the blades appear well-suited to their intended environment. The moderate mechanical properties provide sufficient performance in meat processing, hide work and self-defence, especially in a setting where frequent sharpening and replacement are not feasible as the pastoralists often have to travel great distances to obtain new iron products if there is not a smithy travelling with their encampment [16]. The blades’ low hardness may even serve as an advantage, enabling easier maintenance and greater resilience to fracture.

### 4.4. Cultural and engineering value

Beyond their physical characteristics, the Abarait blades serve as objects of cultural identity and social utility. Their wrist-mounted form, concealed carriage and symbolic status reinforce their embeddedness in Turkana social life. As previous anthropological literature has shown, blacksmiths in many African societies operate not just as toolmakers, but as custodians of ritual, power and tradition [21]. The Abarait, then, should be viewed not merely as a practical implement, but as a physical embodiment of local knowledge systems and values.

The design of the Abarait reflects this dual role. Its elliptical form and central leather-lined aperture make it unobtrusive when worn but immediately functional when needed. This ergonomic design speaks to centuries of refinement under lived conditions, blending concealment with readiness. The engineering of the blade, while lacking industrial precision, demonstrates deep contextual adaptation: a blade that works not in spite of its simplicity, but because of it.

## 5. Conclusions

This project focused on the characterization of the Abarait, a wrist-mounted blade used by the Turkana people of Kenya. Our aim was to investigate its material composition, blade geometry and mechanical properties, and to understand how indigenous forging practices adapted to environmental and cultural needs. To achieve this, two Abarait blades were analysed using three-dimensional scanning, SEM, XRD, XRF and HV30. Sample preparation included segmenting the blades, polishing surfaces and capturing microstructural images for quantitative analysis. Blade sharpness was assessed by measuring edge width and angle from SEM images, and hardness profiles were evaluated across blade sections to infer tensile strength and identify radial trends. Our main results showed that both blades were likely made from low-carbon bloomery iron, featuring significant microstructural variability consistent with traditional smelting and hand-forging processes. XRF identified elements interpreted as natural impurities consistent with the blades being forged from locally sourced iron ore found in the Turkana region’s brackish water or mineral-rich riverbeds. Edge geometry measurements revealed broad cutting angles and considerable variability, favouring durability over precision. Mechanical testing demonstrated moderate hardness and tensile strength values (mean HV30 of 135−150), with statistically significant intra-blade variation but with no radial trend and no evidence of heat treatment. From these findings, we conclude that the Abarait blades were pragmatically engineered to balance toughness and resilience under resource-constrained conditions. The absence of modern metallurgical refinements does not imply inferior quality; rather, it highlights the adaptive ingenuity of Turkana blacksmiths. The blades’ design and material choices were optimized for both utilitarian and defensive functionality, while concurrently embodying cultural identity through their construction and use. While statistically significant differences were identified between the two blades in terms of microstructure, hardness and edge geometry, it is important to note that the sample set of two blades is extremely small, and we do not know the true distribution of the characteristics and properties of Turkana Abarait blades.

## Data Availability

All raw data and processed data is available as supplementary material [41].
